# Development of Visuo-Auditory Integration in Space and Time

**DOI:** 10.3389/fnint.2012.00077

**Published:** 2012-09-17

**Authors:** Monica Gori, Giulio Sandini, David Burr

**Affiliations:** ^1^Robotics, Brain and Cognitive Sciences Department, Istituto Italiano di TecnologiaGenoa, Italy; ^2^Department of Psychology, University of FlorenceFlorence, Italy; ^3^Institute of Neuroscience, National Research CouncilPisa, Italy

**Keywords:** audio, bisection, development, integration, multisensory, space, time, visual

## Abstract

Adults integrate multisensory information optimally (e.g., Ernst and Banks, 2002) while children do not integrate multisensory visual-haptic cues until 8–10 years of age (e.g., Gori et al., 2008). Before that age strong unisensory dominance occurs for size and orientation visual-haptic judgments, possibly reflecting a process of cross-sensory calibration between modalities. It is widely recognized that audition dominates time perception, while vision dominates space perception. Within the framework of the cross-sensory calibration hypothesis, we investigate visual-auditory integration in both space and time with child-friendly spatial and temporal bisection tasks. Unimodal and bimodal (conflictual and not) audio-visual thresholds and PSEs were measured and compared with the Bayesian predictions. In the temporal domain, we found that both in children and adults, audition dominates the bimodal visuo-auditory task both in perceived time and precision thresholds. On the contrary, in the visual-auditory spatial task, children younger than 12 years of age show clear visual dominance (for PSEs), and bimodal thresholds higher than the Bayesian prediction. Only in the adult group did bimodal thresholds become optimal. In agreement with previous studies, our results suggest that also visual-auditory adult-like behavior develops late. We suggest that the visual dominance for space and the auditory dominance for time could reflect a cross-sensory comparison of vision in the spatial visuo-audio task and a cross-sensory comparison of audition in the temporal visuo-audio task.

## Introduction

Multisensory integration is fundamental for our interaction with the world. Many recent studies show that our brain is able to integrate unisensory signals in a statistically optimal fashion, weighting each sense according to its reliability (Clarke and Yuille, 1990; Ghahramani et al., 1997; Ernst and Banks, 2002; Alais and Burr, 2004; Landy et al., 2011). However, children do not integrate unisensory information optimally until late (Gori et al., 2008; Nardini et al., 2008, 2010). We recently showed that in a visual-haptic integration task (similar to that used by Ernst and Banks, 2002) children younger than 8 years of age show unisensory dominance rather than bimodal integration and the modality that dominates is task specific: the haptic modality dominates bimodal size perception and the visual modality dominates orientation bimodal perception (Gori et al., 2008). This dominance could reflect a process of cross-sensory calibration, where in the developing brain the most robust modality is used to calibrate the others (see Burr and Gori, 2011 for a discussion of this idea). It has been suggested that vision calibrates touch for orientation judgments, and touch calibrates vision for size judgments. A good deal of evidence suggests that the calibration process may be fundamental to acquire specific perceptual concepts: in particular we have shown that the impairment of the system that should calibrate the other impacts on the modality that needs calibration (Gori et al., 2010, 2012).

If the communication between sensory modalities has a fundamental role in the development of multisensory function, then we should find different forms of calibration for different dimensions, such as space and time. For example the visual system is the most accurate sense for space judgments and it should be the more influential modality for cross-modal calibration of spatial perception during development. Many studies in adults support this idea, showing that when the spatial locations of audio and visual stimuli are in conflict, vision usually dominates, resulting the so called “ventriloquist effect” (Warren et al., 1981; Mateeff et al., 1985). In adults the ventriloquist effect has been explained as the result of optimal cue-combination where each cue is weighted according to its statistical reliability. Vision dominates perceived location because it specifies location more reliably than audition does (Alais and Burr, 2004). The auditory system, on the other hand, is the most precise sense for temporal judgments (Burr et al., 2009), so it seems reasonable that it should be the more influential in calibrating the perception of temporal aspects of perception during development. In agreement with this idea, studies in adults show that when a flashed spot is accompanied by two beeps, it appears to flash twice (Shams et al., 2000). Furthermore, the apparent multiple flashes actually had lower discrimination thresholds (Berger et al., 2003). Also the apparent frequency of a flickering visual stimulus can be driven up or down by an accompanying auditory stimulus presented at a different rate (Gebhard and Mowbray, 1959; Shipley, 1964), audition dominates in audio-visual time bisection task (Burr et al., 2009), and in general audition seems to affect the interpretation of a visual stimulus also under many other conditions (e.g., see Sekuler and Sekuler, 1999; Shams et al., 2001).

All these results suggest that in the adult visual information has a fundamental role for multisensory space perception, and that audition is fundamental for temporal perception. Like adults, children are immersed in a multisensory world but, as mentioned above, unlike adults they do not integrate optimally across senses until fairly late in development, about 8 years of age (Gori et al., 2008) and some unisensory information seems to be strongly relevant for the creation of specific perceptual aspects (Gori et al., 2008, 2010, 2011; Burr and Gori, 2011; Burr et al., 2011). If the cross-sensory calibration process is necessary for development, then the auditory modality should calibrate vision in a bimodal temporal task, and the visual modality should calibrate audition in a bimodal spatial task. To test this idea we measured visual-auditory integration during development in both the temporal and the spatial domains. To compare the results between the two domains we used a bisection task both in space and in time to study the relative contributions of visual and auditory stimuli to the perceived timing and space of sensory events. For the spatial task we reproduced in 48 children and adults a child-friendly version of the ventriloquist stimuli used by Alais and Burr (2004). For the temporal task we reproduced in 57 children and adults a child-friendly version of the stimulus used by Burr et al. (2009). We also test whether and at which age the relative contributions of vision and audition can be explained by optimal cue-combination (Ernst and Banks, 2002; Alais and Burr, 2004; Landy et al., 2011).

## Materials and Methods

### Audio-visual temporal bisection task

**Figure 1:**
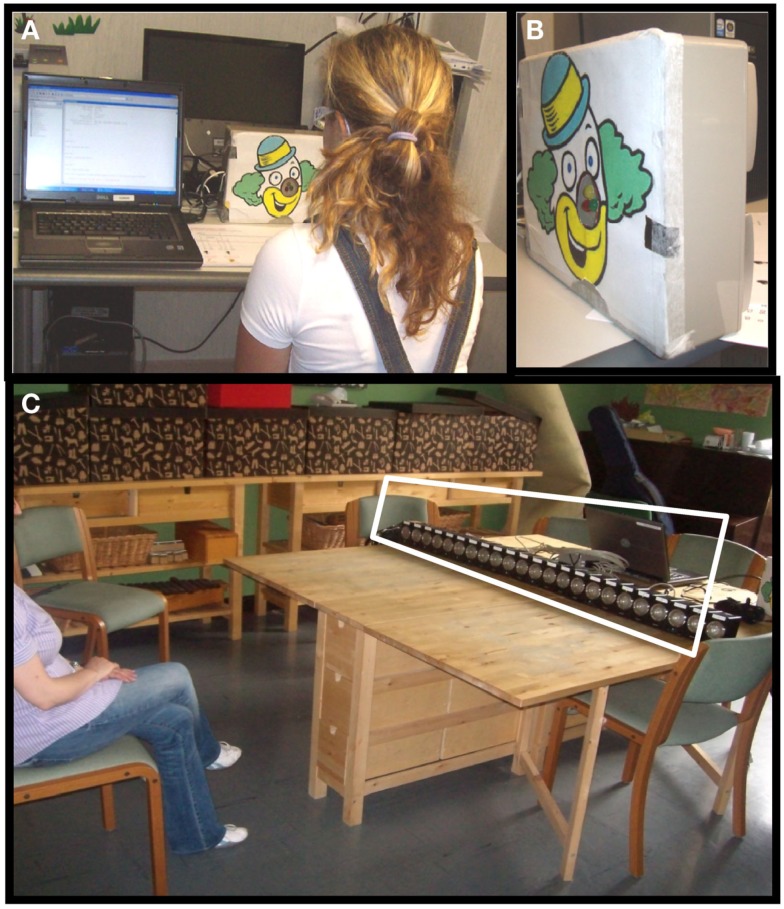
**(A)** Representation of the setup used for the temporal bisection task while a subject is tested. **(B)** Image reporting the setup used for the temporal bisection task. Three lights are presented in front and two speakers are present behind. **(C)** Representation of the setup used for the space bisection task. The blurring panel was positioned in front of the speakers so that the subject could not see the speakers behind it. For illustrative purposes this has been replaced with a transparent panel to show the speakers.

**Figure 2:**
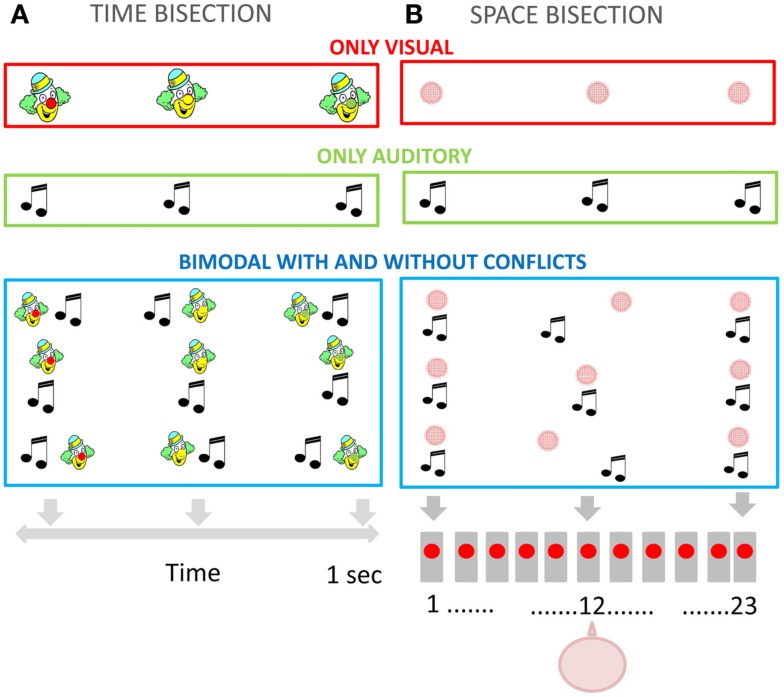
**(A)** Temporal bisection task. Representation of the visual stimulation (upper panel), auditory stimulation (middle panel), and bimodal conflictual and not conflictual visual-auditory stimulation (bottom panel). **(B)** Spatial bisection task. Representation of the visual stimulation (upper panel), auditory stimulation (middle panel), and bimodal conflictual and not conflictual visual-auditory stimulation (bottom panel). The subject was aligned with the speaker in the middle (number 12).

Fifty-seven children and adults performed the unimodal and bimodal temporal bisection tasks (illustrated in Figure 2A). All stimuli were delivered within a child-friendly setup (Figures 1A,B). The child was positioned in front of the setup and observed a sequence of three lights (red, green, and yellow, positioned in the nose of a clown cartoon Figure 1B), listened to a sequence of sounds (produced by speakers spatially aligned with the lights Figure 1B), or both. Three stimuli (visual, auditory, or both) were presented in succession for a total duration of 1000 ms, and the observer reported whether the middle stimulus appeared closer in time to the first or the third stimulus. To help the children to understand the task and the response, they were presented a cartoon with a schematic representation of the two possible responses to be indicated. In the visual task the subject perceived a sequence of three lights: the first one was always red, the second yellow, and the third green. The subject had to respond whether the yellow light appears closer in time to the first or the last one (Figure 2A upper panel). In the auditory task the subject had to respond if the second sound was presented closer in time to the first or the third one (Figure 2A panel in the middle). In the bimodal task the subject perceived a sequence of three lights associated with three sounds (Figure 2A bottom panel). The sequence of the lights presentation was identical to the visual task. The visual and the auditory stimuli could be presented in conflict or not (Δ = −100; 0; 100 ms). The procedure was similar to that used by Burr et al. (2009). In the bimodal condition, all stimuli had an audio-visual conflict, where the auditory stimulus preceded or followed the visual stimulus. For the second stimulus, the conflict was Δ ms (Δ = −50; 0; 50 ms), while for the first and the third stimulus the offset was inverted in sign (-Δ ms).

The visual stimuli were 1°diameter LEDs displayed for 74 ms. Auditory stimuli were tones (750 Hz) displayed for 75 ms. Accurate timing of the visual and auditory stimuli was ensured by setting system priority to maximum during stimulus presentation, avoiding interrupts from other processes (and checking synchrony by recording with microphone and light sensor). The presentation program waited for a frame-synchronization pulse then launched the visual and auditory signals. Before collecting data, subjects were familiarized with the task with two training sessions of 10 trials each (one visual and one audio). Subjects indicated after each presentation of the three stimuli whether the second appeared earlier or later than the midpoint between the first and third stimuli. We provided feedback during these training sessions so observers could learn the task and minimize errors in their responses. No feedback was given after the training sessions. During the experiment proper, five different conditions were intermingled within each session: vision only, auditory only, and three audio-visual conditions. The total single session comprised 150 trials (30 for each condition). The time of presentation of the probe was varied by independent QUEST routines (Watson and Pelli, 1983). Three QUESTs were run simultaneously in the conflict conditions (and one in each of the unisensory conditions). The timing of the second stimulus was adjusted with Quest algorithm (Watson and Pelli, 1983) to home in on the perceived point of bisection of the first and third stimuli. The timing for each trial was given by this quest estimate, plus a random offset drawn from a Gaussian distribution. This procedure ensured that the psychometric function was well sampled at the best point for estimating both the PSE and slope of the functions, as well as giving observers a few “easy” trials from time to time. Also, as the Gaussian offset was centered at zero, it ensured equal responses of closer to first and to third. Data for each condition were fitted by cumulative Gaussians, yielding PSE and threshold estimates from the mean and standard deviation of the best-fitting function, respectively. Standard errors for the PSE and threshold estimates were obtained by bootstrapping (Efron and Tibshirani, 1993). One hundred iterations of bootstrapping were used and the standard error was the standard deviation of the bootstrap distribution. All conflict conditions were used to obtain the two-cue threshold estimates. Both unimodal and bimodal (conflict or not) audio-visual thresholds and PSEs were compared with the prediction of the Bayesian optimal-integration model.

### Audio-visual spatial bisection task

Forty-eight children and adults performed the unimodal and bimodal spatial bisection tasks (illustrated in Figure 2B). Stimuli were presented with a child-friendly setup (Figure 1C) which displayed a sequence of three red light, three sounds, or both. The setup comprised 23 speakers, with a red LED in front of each, which projected onto a white screen in front of the speaker array, yielding a blurred blob of 14° diameter at half height (see Figure 1C). The room was otherwise completely dark. The audio stimulus was identical to that used for the temporal bisection task (see previous section). The subject was seated 75 cm from the screen, causing the speaker array to subtend 102° (each speaker suspended about 4.5°). The child was positioned in front of the central speaker (number 12). Three stimuli (visual, auditory, or both) were presented in succession for a total duration of 1000 ms (identical to the duration used in the temporal bisection task), with the second stimulus occurring always 500 ms after the first. Observers reported whether the middle stimulus appeared closer in space to the first or the third stimulus (corresponding to the speakers at the extreme of the array: see Figure 1C).

In the unisensory visual and auditory task subjects were presented with a sequence of three lights or sounds (Figure 2B upper panel and panel in the middle). In the bimodal task they were presented with a sequence of three lights associated with three sounds (Figure 2B bottom panel). The second stimulus was presented in conflict, the standard now comprised visual and auditory stimuli positioned in different locations: the visual stimulus was the central stimulus +Δ° and the auditory stimulus was the central stimulus −Δ° (Δ = 0 or ±4.5° or ±9°). The first and the last stimuli, the auditory, and visual components were presented aligned, with no spatial conflict. The position of the second stimulus was adjusted with Quest algorithm as for the temporal task. The durations of the auditory and visual stimulations were both 75 ms.

Before collecting data, subjects were familiarized with the task with two training sessions of 10 trials each (one visual and the other audio). To facilitate the understanding of the task and the response in the training phase was presented at the child the image of two monkey cartoons (one red and one green) positioned the red on the left, in proximity of the first speaker and the green on the right, in proximity of the speaker (number 23). The child had to report if the second light was closer to the position of the red or green monkey. Subjects indicated after each presentation of the three stimuli whether the second appeared closer in space to the first or to the third stimulus. We provided feedback during these training sessions so observers could learn the task and minimize errors in their responses. No feedback was given after the training sessions.

During the experiment proper, seven different conditions were intermingled within each session: vision only, auditory only, and five two-cue conditions. The total single session comprised 210 trials (30 for each condition). As before data for each condition were fitted with cumulative Gaussians, yielding PSE and threshold estimates from the mean and standard deviation of the best-fitting function, respectively. Standard errors for the PSE and threshold estimates were obtained by bootstrapping (Efron and Tibshirani, 1993). All conflict conditions were used to obtain the bimodal threshold estimates. Both unimodal and bimodal (conflictual or not) audio-visual thresholds and PSEs were compared with the prediction of the Bayesian optimal-integration model.

In bisection tasks, there are often constant biases, particularly for temporal judgments: the first interval tends to appear longer than the second (Rose and Summers, 1995; Tse et al., 2004). These constant biases were of little interest to the current experiment, so we eliminated them by subtracting from the estimates of each PSE the PSE for the zero conflict condition.

No children with hearing and vision impairments participated to the two tests. We excluded for data recording the children that were not able to perform correctly at least 7 of 10 trials in the training condition (in which the distance between the standard and the comparison were maximal and the test was presented in the simplest version).

### Bayesian predictions

The MLE prediction for the visuo-auditory threshold σ_VA_ is given by:

(1) $$\sigma_{\text{VA}}^{2}=\frac{\sigma_{\text{V}}^{2}\sigma_{\text{A}}^{2}}{\sigma_{\text{V}}^{2}+\sigma_{\text{A}}^{2}}\leq \min\left(\sigma_{\text{V}}^{2},\sigma_{\text{A}}^{2}\right)$$

where σ_V_ and σ_A_ are the visual and auditory unimodal thresholds. The improvement is greatest (√2) when σ_V_= σ_A_.

The MLE calculation assumes also that for time and space judgments, the optimal bimodal estimate of PSE $\left({Ŝ}_{\text{AV}}\right)$ is given by the weighted sum of the independent audio and visual estimates $\left({Ŝ}_{\text{V}}\;\text{and}\;{Ŝ}_{\text{A}}\right)$.

(2) $${Ŝ}_{\text{VA}}=w_{\text{V}}{Ŝ}_{\text{V}}+w_{\text{A}}{Ŝ}_{\text{A}}$$

Where weights *w*_V_ and *w*_A_ sum to unity and are inversely proportional to the variance (σ^2^) of the underlying noise distribution, assessed from the standard deviation σ of the Gaussian fit of the psychometric functions for visual and auditory judgments:

(3) $$w_{\text{V}}=\frac{\sigma_{\text{A}}^{\text{2}}}{\left(\sigma_{\text{A}}^{\text{2}}+\sigma_{\text{V}}^{\text{2}}\right)},\;w_{\text{A}}=\frac{\sigma_{\text{V}}^{\text{2}}}{\left(\sigma_{\text{A}}^{\text{2}}+\sigma_{\text{V}}^{\text{2}}\right)}$$

To calculate the visual and auditory weights from the PSEs (Figure 6), we substituted the actual spaces or times (relative to standard) into Eq. 2:

(4) $$Ŝ\left(\Delta \right)=\left(w_{\text{V}}\Delta -w_{\text{A}}\Delta \right)=\left(1-2w_{\text{A}}\right)\Delta$$

The slope of the function is given by the first derivative:

(5) $$Ŝ{\left(\Delta \right)}^{\prime }=1-2w_{\text{A}}$$

Rearranging:

(6) $$w_{\text{A}}=\frac{\left(1-Ŝ{\left(\Delta \right)}^{\prime }\right)}{2}$$

The slope $Ŝ{\left(\Delta \right)}^{\prime }$ was calculated by linear regression of PSEs for all values of Δ, separately for each child and each condition.

The data of Figure 5 show as a function of age the proportion of the variance of the PSE data explained by the MLE model. The explained variance *R*^2^ was calculated by:

(7) $$R^{2}=1-\frac{1}{{\hat{\sigma }}^{2}+\sigma^{2}}\cdot \frac{1}{N}\cdot^{\overset{N}{\underset{i=1}{\sum }}{\left(S_{i}-{Ŝ}_{i}\right)}^{2}}$$

Where *N* is the total number of PSE values for each specific age group (all children and all values of Δ), *S_i_* the individual PSEs for time and space, ${Ŝ}_{i}$ is the predicted PSE for each specific condition, ${\hat{\sigma }}^{2}$ is the variance associated with the predicted PSEs and σ^2^ the variance associated with the measured PSEs. *R*^2 ^= 1 implies that the model explains all the variance of the data, *R*^2 ^= 0 implies that it does no better (or worse) than the mean, and *R*^2 ^< 0 implies that the model is worse than the mean.

## Results

**Figure 3:**
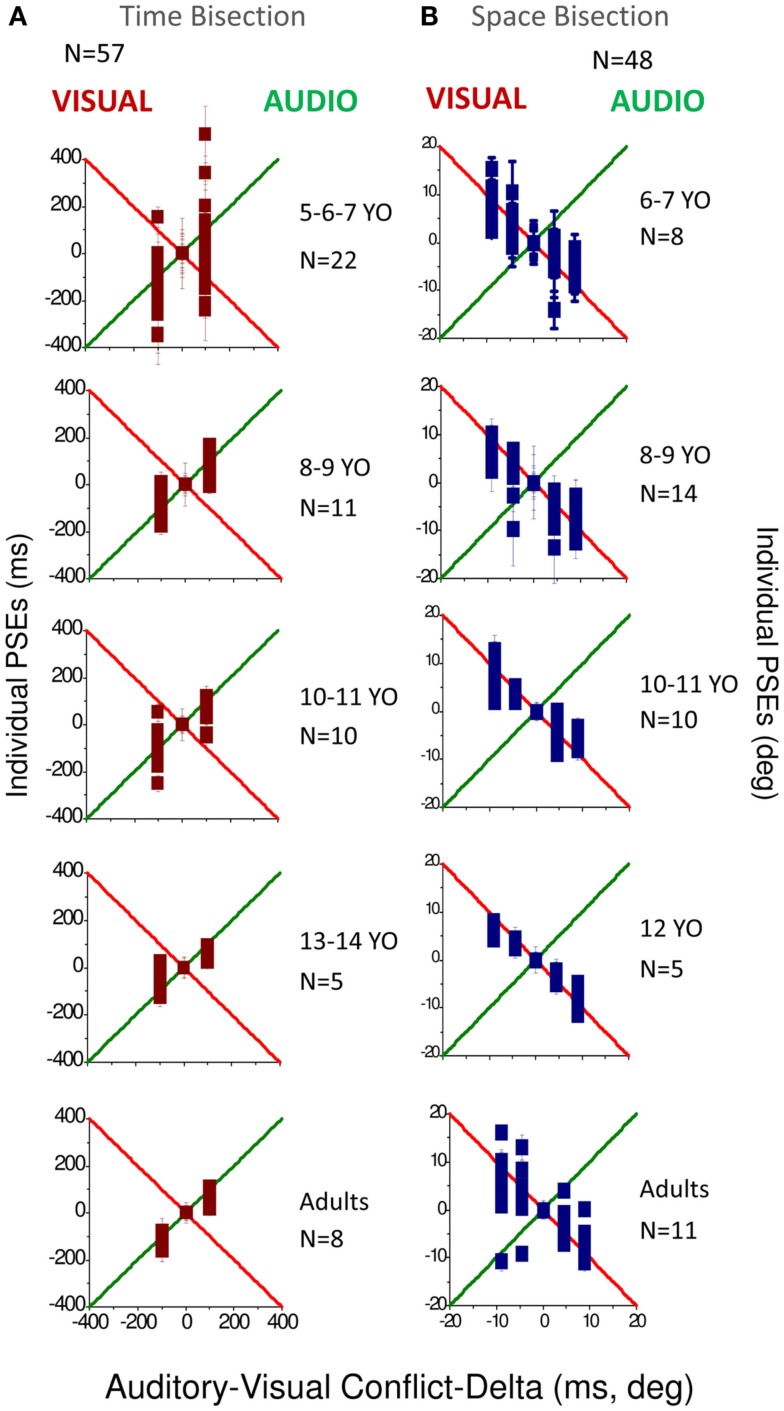
**(A)** PSEs measured for the different conflictual condition in the temporal bisection task. **(B)** PSEs measured for the different conflictual condition in the spatial bisection task. In both panels the green line represents total auditory dominance and the red line total visual dominance. Different ages are reported in different panels. The number of subjects who participated is indicated in each panel for each age and condition.

Figure 3 reports the PSEs for both temporal bisection (Figure 3A) and space bisection (Figure 3B). In both Figures we adjusted the PSEs for constant errors in bias by subtracting for each conflictual PSE the PSE obtained in the not conflictual condition. In the temporal bisection task (Figure 3A), PSEs tend to follow the green line, suggesting auditory dominance over vision. As may be expected, the results for the 5–7 age-group are noisier than the others, but the tendency is similar at all ages, particularly the older age-groups. In the audio-visual spatial bisection task (Figure 3B) PSEs follow the visual standard (indicated by the red line) especially until 12 years of age.

**Figure 4:**
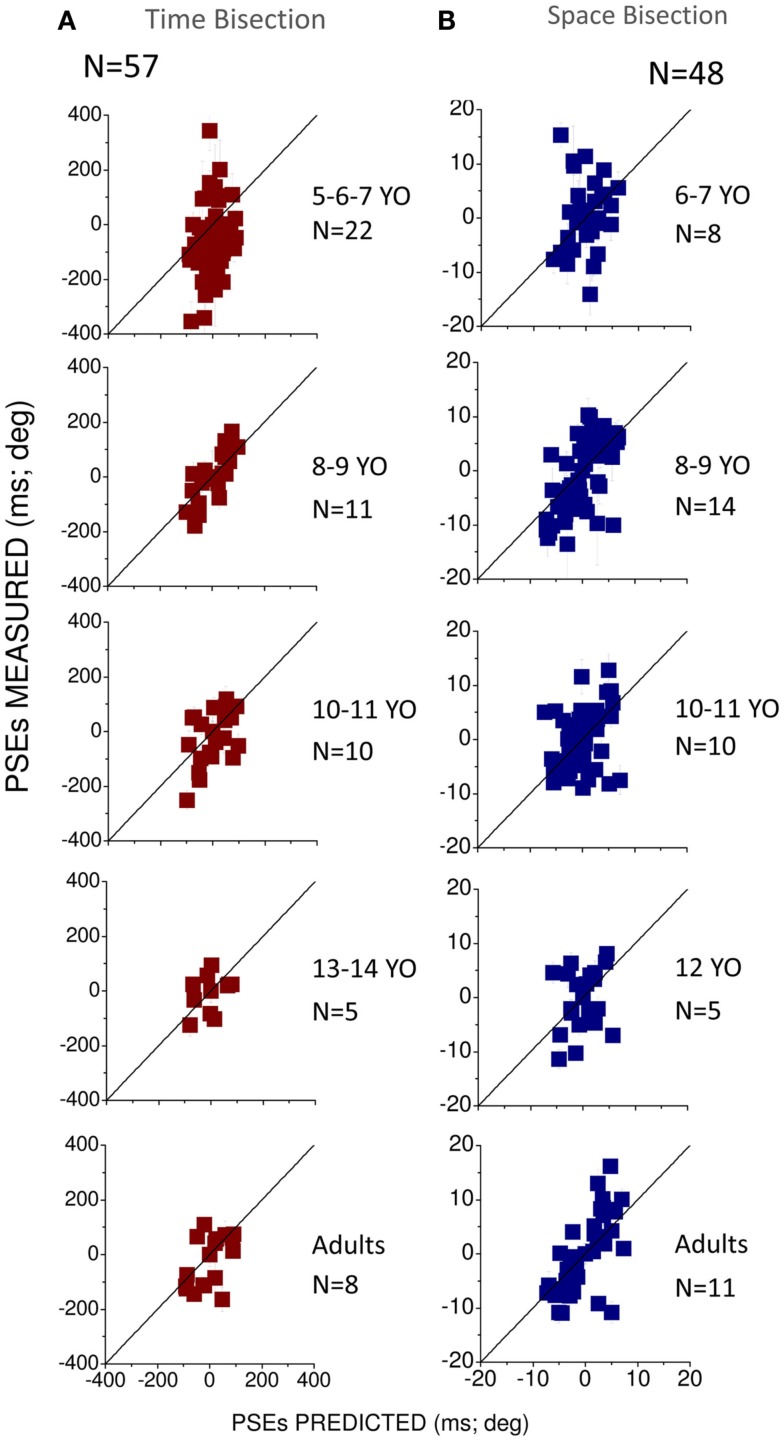
**(A)** Measured against predicted PSEs for the different conflictual conditions in the temporal bisection task. **(B)** Measured against predicted PSEs for the different conflictual condition in the spatial bisection task. In both panels the black line represents the prediction of the Bayesian model and suggests optimal integration. Different ages are reported in different panels. The number of subjects who participated is indicated in each panel for each age and condition.

To observe how much this behavior is predicted by the MLE model, we plotted in Figures 4A,B the PSEs measured against the PSEs predicted by the Bayesian model (Eq. 2). Superimposition of the dots on the black line (equality line) would suggest that the behavior of the group is well predicted by the Bayesian model. From this graph we can observe that for the temporal bisection task (Figure 4A) the behavior becomes adult-like at about 8–9 years of age when the dots lie close to (but not entirely superimposed on) the equality black line as occurs in the adult groups. On the other hand, for the space bisection task, the dots lie on the equality line only in the adult group (Figure 4B).

**Figure 5:**
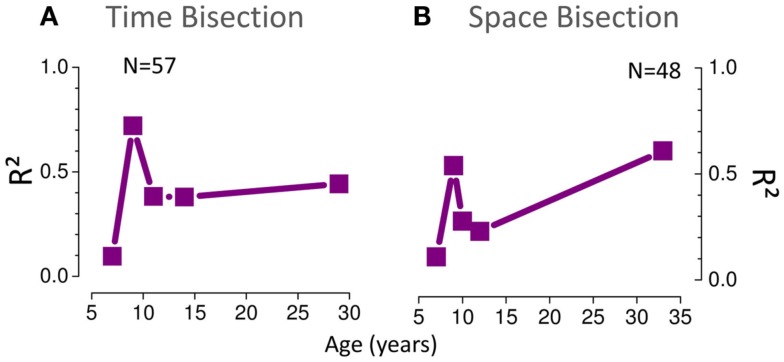
**(A)** Proportion of variance (*R*^2^) of the PSE data (Figure 3) for the time bisection task explained by the MLE model. A value of 1 means that all the variance was explained by the model, 0 that the model performed as well as the mean, and less than 0 that it performed worse than the mean (see Eq. 7). **(B)** The same for the space bisection task.

Figure 5 summarizes how visuo-auditory integration develops with age. It plots the amount of variance (*R*^2^) in PSEs explained by MLE model. A value of 1 means that all the variance was explained by the model, 0 that the model performed as well as the mean, and less than 0 that it performed worse than the mean (see Eq. 7). For both the spatial and temporal tasks, the MLE model explains a large proportion of the variance at all ages except the youngest (6-year-olds). For both space and time in the 6 years old group *R*^2^ ≃ 0, suggesting that the model performed as well as the mean. The 8-year-old group shows a larger proportion of explained variance (*R*^2^ > 0.5) but interestingly, there is a dip in the curve at 10–12 years showing less explained variance, especially for the space bisection test (*R*^2^ < 0.5). In the adult group a larger amount of variance is explained by the MLE model in the space bisection task than in the time bisection task suggesting better integration for the first task.

**Figure 6:**
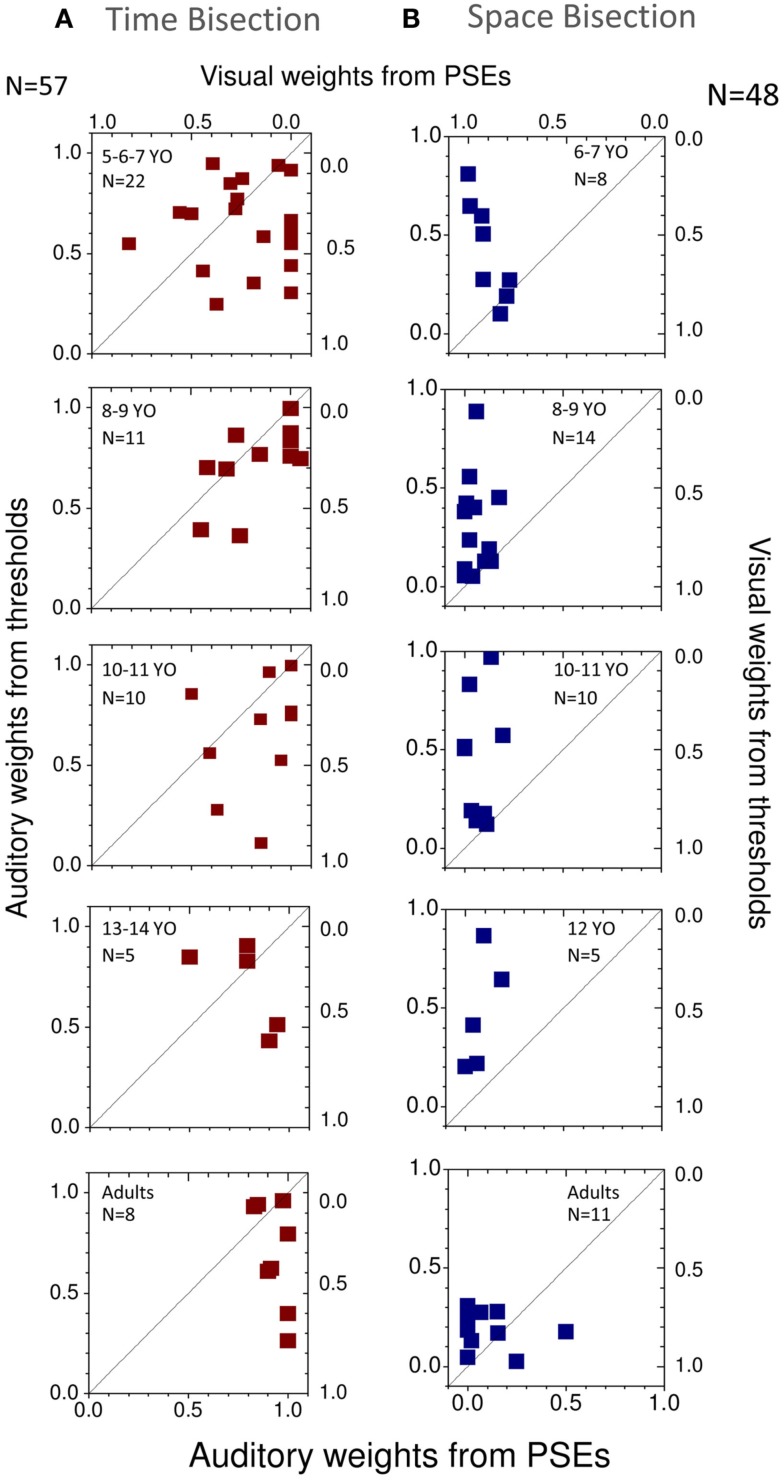
**(A)** Individual weights predicted from thresholds plotted against those predicted from PSEs for different ages for the time bisection task. The black line shows the equality line. **(B)** Same for the space bisection task.

We then calculated the audio and visual weights required for the Bayesian sum (Eq. 2), separately from the estimates of PSEs (Eqs 4–6) and from the estimates of unimodal thresholds (Eq. 3). The results are plotted in Figure 6, showing auditory weights on the left ordinate and visual weights on the right (the two sum to unity). In general, for the time bisection (Figure 6A), the auditory weight for the PSE was more than that predicted by thresholds (points tend to fall to the right of the bisector). This occurred at all ages, but was clearest for the adults. Conversely, for the space bisection (Figure 6B), the PSE has less auditory weight (more visual weight) than predicted by thresholds until adulthood.

**Figure 7:**
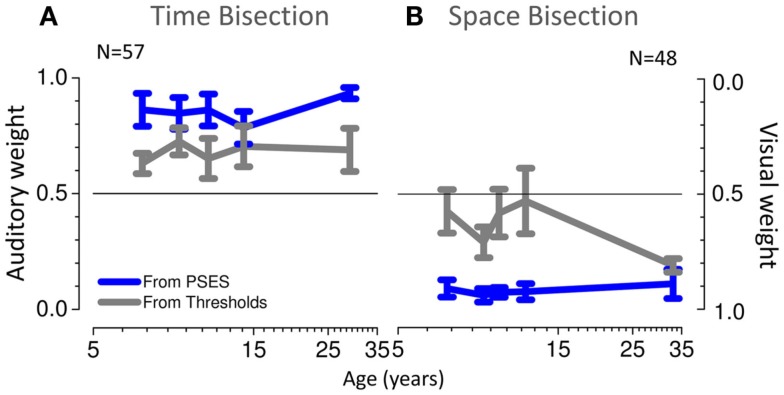
**(A)** Average weights as a function of age, predicted from thresholds in gray and from PSEs in blue, for the time bisection task. **(B)** Same for the space bisection task.

Figure 7 plots average theoretical auditory and visual weights as a function of age: gray lines show the MLE-predicted weights (Eq. 3), and blue lines the weights calculated from the PSE vs. conflict functions (Eq. 6). These graphs tell a similar story to Figure 6. For temporal judgments (Figure 7A), the PSEs show a greater auditory weight than predicted by thresholds while for spatial judgments (Figure 7B) the PSEs show a greater visual weight than predicted. The only exception is the spatial judgments for adults, where PSE and thresholds estimates are very similar (both heavily biased toward vision).

**Figure 8:**
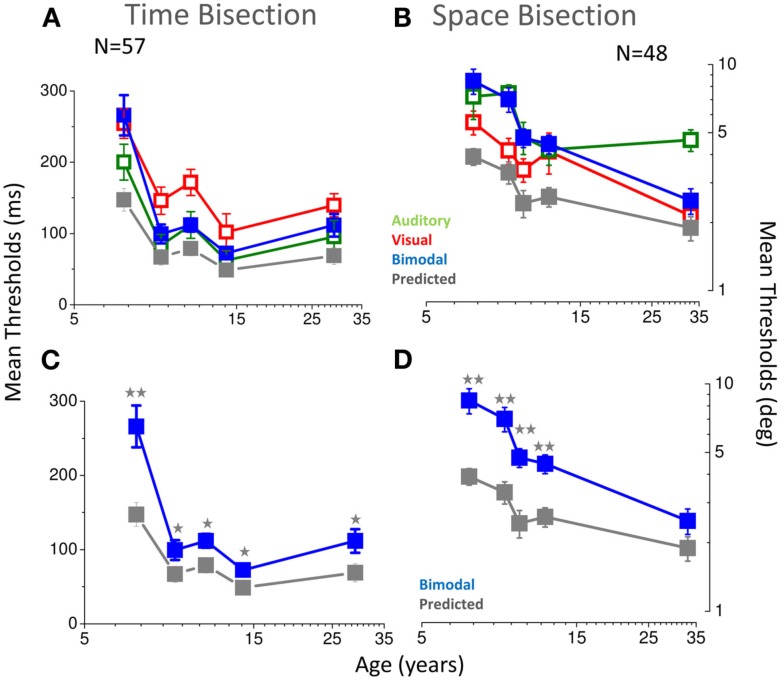
**(A)** Thresholds as a function of age for the temporal bisection task. Visual thresholds are reported in red, auditory in green, bimodal in blue, and predictions of the Bayesian model in gray. **(B)** Same for the space bisection task. **(C)** Same as A, showing for clarity only bimodal thresholds (blue) and Bayesian prediction (gray). **(D)** Same as C for the space bisection task. In all cases, two stars represent a significance level of less than 0.01 and one star a significance level of less than 0.05 in a one tailed one sample *t*-tests.

The strong test of optimal integration is an improvement in bimodal thresholds (given by the standard deviation of the cumulative Gaussian fits). Figure 8 shows the results. For the temporal bisection task (blue dots in Figures 8A–C), the improvement in thresholds for bimodal presentations was less than predicted at all ages (see stars in Figure 8C and caption), if compared with the Bayesian prediction (gray symbols in Figures 8A–C). In the youngest group of children (5–7 years of age), bimodal thresholds follow the poorer modality (the visual one, red and blue dots in Figure 8A). Interestingly, at this age the bimodal PSEs also are much noisier than the older groups (see Figure 4A). After 7 years of age, when also PSEs become less noisy and adult-like, bimodal thresholds become identical to the auditory thresholds and remain equal to the auditory one also in the older groups (green dots in Figure 8A). Also for the space bisection task, PSEs and thresholds show related behaviors: when PSEs show less inter-subject variability (in the adult group), the bimodal thresholds become well predicted by the Bayesian model (blue and gray dots in Figure 8B, see stars in Figure 8D). In the younger groups they follow the poorer sense (the auditory one, blue and green dots in Figure 8B).

## Discussion

### Audio-visual space and time bisection in adults

In this study we investigated audio-visual integration in space and in time perception during development. The goal was to examine the roles of the visual and auditory systems in the development of spatial and temporal aspects. To compare these two aspects, similar tasks were used to study space and time, requiring subjects to bisect temporal or spatial intervals. In adults, optimal multisensory integration, which has been reported for many tasks (Clarke and Yuille, 1990; Ghahramani et al., 1997; Ernst and Banks, 2002; Alais and Burr, 2004; Landy et al., 2011), is not evident in our temporal bisection task at any age tested and is evident in our spatial bimodal task only for the adult group. The absence of integration obtained in our temporal task is in agreement with other studies (e.g., Tomassini et al., 2011) that show that multisensory integration is sub-optimal also for a visual-tactile time reproduction tasks. It is also in agreement with previous studies that show auditory dominance over vision rather than optimal integration in adults (Shams et al., 2000; Burr et al., 2009) for temporal localization. In particular, Burr et al. (2009) examined audio-visual integration in adults using a bisection task (similar to the one we used), and found that sound does tend to dominate the perceived timing of audio-visual stimuli. Our stimulus is for the most part similar to the stimulus used by Burr et al. (2009) with few exceptions. One difference was the larger temporal conflicts and the fact that all the three stimuli presented in the conflictual conditions contained conflict information, while in the Burr et al. (2009) stimuli the conflict was only in the first and last stimuli. Overall, if some differences between these two experiments were present, our results are mostly in agreement with those of Burr et al. (2009), particularly for the fact that auditory dominance of PSEs was not well predicted by the Bayesian model, with more weight to audition than predicted from thresholds. This audio dominance can be specific to the audio stimulus used. Burr et al. (2009) reported that bimodal prediction of thresholds was less successful for higher auditory tones (1700 Hz) than for lower tones (200 Hz) and in agreement with this finding we found auditory dominance rather than optimal integration by using a high auditory tone (750 Hz).

Our results on audio-visual space integration in adults agree well with previous studies. Like Alais and Burr (2004), we found optimal integration of bimodal thresholds, shown by an increment in precision compared with the unisensory performances. Both visual and multisensory thresholds (considering a similar visual blurred condition) were similar to those obtained by Alais and Burr (2004). Our auditory thresholds were better than those obtained by Alais and Burr (2004), possibly because of the different audio stimulation. Indeed in their experiment the audio stimulus was defined by only one cue (interaural timing difference), while our stimuli were real speakers in space, thereby providing many cues to localization, binaural and monaural. On the other hand our results suggest sub-optimal integration for PSEs, for which the proportion of the variance of the PSEs data is not completely explained by the MLE model (see Figure 5) and the weights predicted from thresholds are not completely superimposed to those computed from PSEs (see Figure 7). A possible explanation for this difference could be that the task in our experiment was a bisection task rather than the discrimination task as used by Alais and Burr (2004). Another difference could be that Alais and Burr’s subjects were trained extensively on the auditory task and were instructed to attend to both visual and auditory aspects of the stimuli. Given the limited time available to test children (and not wanting differences between children and adults), all subjects had the same 20 trials of training without particular attention to the auditory or bimodal aspects.

### Audio-visual space and time bisection in children

In agreement with our previous results (Gori et al., 2008), we found that for both tasks the bimodal adult-like behavior emerges only late in development. For the time bisection the adult-like behavior occurs after 8 years of age while for the space bisection task, it was fully mature only in our adult group. Like the visual-haptic studies (Gori et al., 2008), children show strong unisensory dominance rather than multisensory integration of audio and visual space and time perception. In the child, audition dominates visual-auditory time perception and vision dominates visual-auditory space perception. This result is in agreement with our prediction and in line with our cross-sensory calibration theory (Burr and Gori, 2011). The auditory dominance can reflect a process of cross-sensory calibration in which the auditory system could be used to calibrate the visual sense of time since it is the most accurate sense for temporal judgments. This result is also in agreement with many experiments performed with adults that show a dominant role of the auditory system for time (Gebhard and Mowbray, 1959; Sekuler and Sekuler, 1999; Shams et al., 2000, 2001; Berger et al., 2003; Burr et al., 2009). Why the auditory dominance of both PSEs and bimodal thresholds persists into adulthood is not clear. A possible explanation is that for this kind of task the cross-sensory calibration process is still occurring since audition is too accurate with respect to the visual modality, and the precision of the visual system for this kind of task prevents the transition from unisensory dominance to multisensory integration. This dominance may however not be apparent with a different kind of stimulation. For example it would be interesting to observe whether auditory dominance in children occurs in other visual-auditory temporal integration tasks for which a strong multisensory integration in adults has been reported (as for example reducing the auditory tone from 750 to 200 Hz).

Similarly, the visual dominance of space during development could reflect a process of cross-sensory calibration in which the visual system is used to calibrate the auditory system for space perception, since it is the most accurate spatial sense. In agreement with this idea, many studies in adults show that the visual system is the most influential in determining the apparent spatial position of auditory stimuli (Pick et al., 1969; Warren et al., 1981; Mateeff et al., 1985; Alais and Burr, 2004). Only after 12 years of age, visual-auditory integration seems to occur in this spatial task suggesting a very late development. Audio-visual space integration seems to mature later than visual-haptic spatial integration (that develops after 8–10 years of age, Gori et al., 2008) and also visual-auditory temporal integration. This could be related to the time of maturation of the individual sensory systems. Indeed, our previous work (Gori et al., 2008) suggested that multisensory integration occurs after the maturation of each unisensory system. The unisensory thresholds of Figure 8 suggest that both visual and auditory thresholds continue to improve over the school years, particularly for the spatial task. For the space bisection task, the unisensory thresholds are still not mature at 12 years of age, and nor is integration optimal at this age. For the temporal task, unisensory thresholds become adult-like after 8–9 years of age, and at this age the auditory dominance appears. A delay in the development of unisensory systems seems to be related to the delay in the development of multisensory adult-like behavior.

These results support the idea that in children the use of one sense to calibrate the other precludes useful combination of the two sources (Gori et al., 2008; Burr and Gori, 2011). On the other hand, given the strong variability between subjects and also the noise in the developing system we cannot exclude the possibility that these results reflect the greater noise in the sensory system of the developing child. The fact that the weights derived from thresholds lie at the midpoint between auditory and visual dominance do not allow us to exclude this hypothesis.

To examine further whether this dominance reflects a process of cross-sensory calibration it would be interesting to measure how the impairment of the dominant system impacts on the non-dominant modality that may need calibration (as we did in Gori et al., 2010, 2012). In particular, it would be interesting to see how auditory spatial perception is impaired in children and adults with visual disabilities and how visual time perception is impaired in children and adults with auditory disabilities by using stimuli and procedures similar to those used in this study. If this dominance really reflects a process of a cross-sensory calibration it should allow clear and important predictions about spatial and temporal deficits in children and adults with visual and auditory disabilities.

## Conflict of Interest Statement

The authors declare that the research was conducted in the absence of any commercial or financial relationships that could be construed as a potential conflict of interest.
